# Smokefree signage at children’s playgrounds: Field observations and comparison with Google Street View

**DOI:** 10.1186/s12971-017-0143-x

**Published:** 2017-08-23

**Authors:** George Thomson, Nick Wilson

**Affiliations:** University of Otago, Wellington, New Zealand

**Keywords:** Children, Playgrounds, Health communication, Health promotion, Outdoor field observation, Google Street View

## Abstract

**Background:**

Although there is global growth in outdoor smokefree areas, little is known about the associated smokefree signage. We aimed to study smokefree signage at playgrounds and to compare field observations with images from Google Street View (GSV).

**Methods:**

We randomly selected playgrounds in 21 contiguous local government areas in the lower North Island of New Zealand, all of which had smokefree playground policies. Field data were collected on smokefree signage along with dog control signage to allow for comparisons. The sensitivity and specificity of using GSV for data collection were calculated.

**Results:**

Out of the 63 playgrounds studied, only 44% (95% CI: 33%–57%) had any smokefree signage within 10 m of the playground equipment. The mean number of such signs was 0.8 per playground (range: 0 to 6). Sign size varied greatly from 42 cm^2^ up to 2880 cm^2^; but was typically fairly small (median = 600 cm^2^; ie, as per a 20 × 30 cm rectangle). Qualitatively the dog signs appeared to use clearer images and were less wordy than the smokefree signs.

Most playground equipment (82%), could be seen on GSV, but for these settings the sensitivity for identifying smokefree signs was poor at 16%. Yet specificity was reasonable at 96%.

**Conclusions:**

The presence and quality of smokefree signage was poor in this sample of children’s playgrounds in this developed country setting. There appears to be value in comparing smokefree signage with other types of signage (eg, dog control signage). Google Street View was not a sensitive tool for studying such signage.

**Electronic supplementary material:**

The online version of this article (10.1186/s12971-017-0143-x) contains supplementary material, which is available to authorized users.

## Background

Outdoor smokefree area policies are expanding internationally [1], and include such settings as outdoor eating/drinking areas at bars and restaurants, school and hospital grounds, beaches, parks, and playgrounds. There is some evidence that such policies are effective [2–5], although variable compliance has been described for some (eg, [6]). However, there is relatively little research on the signage in any such settings. Such work focuses predominantly on sign prevalence, using either convenience samples or single jurisdictions [7–10]. Our previous related publications used convenience samples [8], focused on smokefree school grounds signs [7], on hospital signs [11], and, for smokefree signage at a range of types of places, on the contrasts between suburbs [9]. Given this background, we aimed to study smokefree signage at a random sample of publically accessible children’s playgrounds in New Zealand, and to estimate the utility of using Google Street View (GSV) to collect such data. Because of the lower observation cost and wide international coverage, GSV is increasingly being used in health research (as per a recent review [7, 11, 12]). New Zealand local government Territorial Local Authorities (TLAs) can have smokefree policies for their playgrounds, but at present do not use bylaws (ordinances) to enforce such policies.

## Methods

### Sampling frame

We searched for public playgrounds on TLA website listings of parks and playgrounds in 21 contiguous TLA areas in the lower North Island of New Zealand (of which 10 encompassed cities and 11 were largely rural) see Additional file 1: Table S1, *Online Appendix*. Thus the sample excluded playgrounds in school or pre-school grounds, which are legally required to be smokefree [2]. Where playground lists were not available (*n* = 5), we compiled them ourselves by identifying playgrounds from the satellite view in Google Maps in all the parks in the TLA area. The 21 TLAs ranged from one of the most deprived and smallest (Wairoa) to one of the least deprived (Wellington) and all had smokefree policies for playgrounds. The policies are for all the area of the playgrounds, and are not defined by the distance to playground equipment. In each TLA we randomly selected either two playgrounds or a 10% sample of all the playgrounds, whichever was the larger number (ie, up to 11 for Wellington City) for a total of 63. Playgrounds were defined as having fixed play equipment (swings, slides etc) or a skate park.

### Field data

Photographs were taken of all smokefree signage within 10 m of the playground equipment and the largest sign was measured with a tape measure. The distance of 10 m was chosen because sign perception is a product of distance and the features of signs, including size and readability [13]. From preliminary field observations, we estimated that a typical playground sign that is 30 cm square should be visible at 10 m, even with poor text and graphic design. Ten metres is also the normal distance from playground equipment specified by Australian states for smokefree policies.

Our previous work, using a convenience sample of 54 playgrounds [8], recorded smokefree signage at the entrance of paths leading playgrounds, and (where the playgrounds were in parks) within 100 m of the equipment. However, as we were aiming to develop a simple method that recorded the *most* effective signs for playgrounds, we focused only on those within 10 m of the equipment. We considered that as playgrounds usually have several or many paths leading to them, or are surrounded by grass, path entrance signs (where present) would not substitute for those within 10 m of equipment. Similarly, smokefree park signs can be distant to the playgrounds, and we considered that they would be less effective for a smokefree playground policy.

As in previous work, the observers (NW, GT) made trial observations and then agreed on a data collection protocol that was sufficiently uniform and effective. A number of photos were taken to provide both close-up detail and wider context for the visible signs. The observers walked around the playgrounds, taking photos from all sides, to ensure comprehensive coverage.

For comparison purposes, the same data were collected on dog control signs (including permissive signage allowing dog walking). Data collection occurred during September – December 2016 (by one or both authors).

### Google Street View data

The playgrounds were examined using GSV and the visibility of signage documented. The approach taken to calculating sensitivity and specificity was the same as used in a previous signage study where the “gold standard” was assumed to be the field observation data [7]. We use the term ‘sensitivity’ as the ability of GSV observation to correctly detect the presence of signs (the true positive rate), and ‘specificity’ as the ability to correctly detect their absence (the true negative rate). An independent observer (see Acknowledgements) who was not involved in the field work collected the GSV data.

## Results

Out of the 63 playgrounds studied, only 44% (95% CI: 33%–57%) had smokefree signage within 10 m of the playground equipment (Table 1). This proportion was slightly higher in cities than in other TLA areas (49% vs 38%), but this difference was not statistically significant. The mean number of such signs was only 0.8 per playground (range: 0 to 6), even though some playgrounds had multiple paths leading to them. Results for each of the 21 TLAs are given in the *Online Appendix*.

**Table 1:** Smokefree signage and dog control signage at 63 children’s playgrounds in 21 contiguous local government authority areas in the lower North Island of New Zealand (for signs within 10 m of the playground equipment)

Characteristic	Smokefree signs [95% CI]	Dog control signs [95% CI]	*P*-value for difference
Playgrounds with any sign (N)	28	20	–
% of all playgrounds with any sign	44% (28/63)	32% (20/63)	0.149
Local government areas with any signs (*n* = 21 areas)	71%	48%	0.133
Playgrounds in city areas (*n* = 39) with signs	49%	36%	0.264
Playgrounds in other local government areas (*n* = 24)	38%	25%	0.373
Total signs (ie, multiple signs in some playgrounds)	50	28	–
Mean number of signs per playground (SD)	0.79 (1.23)	0.44 (0.80)	0.095
Range for signs per playground (N)	0 to 6	0 to 4	–
*Sign size*			
Average in cm^2^ (SD)	1057 (960)	1154 (1161)	0.749
Median in cm^2^	600	768	–
Inter-quartile range (cm^2^)	233 to 1800	440 to 1350	–
Full range (cm^2^)	42 to 2880	100 to 3600	–
*Performance of GSV (using field observations as the gold standard)*			
Play equipment not visible on GSV (too far from a road) – these results excluded from further analysis	11 (17.5%)	11 (17.5%)	–
True positives [A]	4	7	–
True negatives [B]	26	32	–
False positives [C]^a^	1	3	–
False negatives [D]	21	10	–
Sensitivity [A/(A + D)]	16% [5% – 34%]	41% [20% – 65%]	0.087
Specificity [B/(B + C)]	96% [83% – 100%]	91% [78% – 98%]	0.505
Positive predictive value [A/(A + C)]	80% [33% – 99%]	70% [38% – 92%]	0.747
Negative predictive value [B/(B + D)]	55% [41% – 69%]	76% [62% – 87%]	0.043

^a^ These “false positives” may be a true error (the GSV user mis-interpreting another sign eg, a “no horses” sign for a “no dogs” sign) or may actually reflect a smokefree or dog control sign that was previously present when the GSV image was taken several years ago, but was not present in the field observations in 2016

Sign size varied from 42 cm^2^ (equivalent to a 6 cm × 7 cm rectangle) up to 2880 cm^2^ (equivalent to a 50 cm by 58 cm rectangle); but was typically fairly small (median = 600 cm^2^; ie, as per a 20 × 30 cm rectangle). Even with small signs, often much of the sign surface was white space (eg, Additional file 1: Fig. A1 in the *Online Appendix*). There were at least 15 different styles of smokefree sign design (apart from size differences) with style differences even within TLAs.

There were no statistically significant quantitative differences between the smokefree and dog control signage at the playgrounds, though the average and median sizes of the dog signs was slightly larger than the smokefree signs (Table 1). Qualitatively the dog signs appeared to be less wordy than the smokefree signs and often simply had a symbol of a dog crossed out (Additional file 1: Figs. A1 and A2). Dog control signage also appeared to be more directive, with use of such words as “prohibited” (Additional file 1: Fig. A2). Such words were never used in any smokefree signs. Smokefree signage appeared to be more explanatory in the sense of conveying air quality messages (eg, “fresh air”), and for healthy role modelling around children (“we copy what we see” in a messages to adults, Additional file 1: Figs. A2 and A3). Both types of signs were very diverse in style and content, with some including the names of the local area and Māori (indigenous) language (Additional file 1: Fig. A4).

The equipment in most of the playgrounds (82%) could be seen on GSV, but for these settings the sensitivity for identifying smokefree signs was poor at 16% (Table 1). Specificity was reasonable at 96%. The results for dog control signage were somewhat better for sensitivity (41%) and similar for specificity (91%).

## Discussion

The range of TLAs provided different styles of signage, signage use, and demographic context. We found a majority of playgrounds without smokefree signage, and a small median size where there were signs.

There appears to be value in comparing smokefree signage with other types of signage (eg, dog control signage), especially in terms of qualitative aspects. More formal semiotic analysis of the signs could be considered by marketing and communication experts, but our impression is that there is large scope for improvements in sign design. Internationally, there is a lack of research on the effective elements for smokefree signs, with one of the few articles being from 1981. This indicated that positive rather than negative messages may be more effective [14]. Smoker perceptions that smoking is not normal appear to be associated with quitting and quit success [15, 16]. Thus the use of denormalisation messages that focus on modelling to children may also be an effective part of media to help smokers quit, as well as helping protect children from smoking normalisation [17]. The phrase used in some New Zealand signs ‘we copy what we see’ echoes the words ‘children see, children do’ used in some North American smokefree media.

While GSV appears to have some utility for studying other types of smokefree signage [7, 11], it was not useful for these playground signs, with low sensitivity. This problem was because: (i) some playgrounds were visible but distant from the road; (ii) some signs were quite small and not legible in the GSV imagery; and (iii) the GSV imagery was sometimes a few years out-of-date (ie, signage observed in the field observations may have been installed subsequently). But use of GSV for monitoring such signage could be reconsidered in future studies, as GSV continues to develop (eg, expanding use of “footpath views”).

Future research could also use larger samples per jurisdiction, and be across a greater number of jurisdictions, so as to provide better statistical power and generalisability.

### Policy implications

Signage is important in communicating policies, whether the policies are educational (seeking to persuade without legal backing) or are enforceable. They need to be salient and comprehensible, and in conjunction with communication by other media, achieve an effective response. However, in the absence of other effective communication, where smokefree policies (backed by regulations) are not effectively enforced, or the smokefree policies have no legal backing, signs are crucial as being the main avenue of policy implementation. All countries should ideally provide guidelines on such signage, so that citizens recognise the same message across sub-national jurisdictions. Such guidelines should be research-based, and may improve the effectiveness of such signs. Ideally, governments could provide well-designed signs for free (as they do for school and pre-school grounds in New Zealand where they are legally mandated [2]). Furthermore, signage should ideally be part of a wider strategy to communicate smokefree or tobacco-free messages and norms, and may be one component of a national comprehensive tobacco control strategy [18].

## Conclusions

This appears to be the first study of smokefree signs at randomly selected playgrounds in many jurisdictions. While there were no significant quantitative differences found relative to dog control signage, qualitatively the dog signs appeared to use clearer images and were less wordy than the smokefree signs. Google Street View has not previously been used to study smokefree signage at children’s playgrounds. We found it to be poor for the purpose.

## Additional file

Additional file 1: Online appendix. (PDF 1085 kb)

## Abbreviations

GSV: Goggle Street View
TLA: Territorial Local Authority
