# Supplementary material for: Smokefree signage at children’s playgrounds: Field observations and comparison with Google Street View
Source: Tob Induc Dis. 2017 Aug 23;15:37. doi: 10.1186/s12971-017-0143-x (PMC5569489; doi:10.1186/s12971-017-0143-x)

ONLINE APPENDIX

Table A1. Local government area specific results for smokefree and dog control signage (at 63 children's playgrounds in 21 contiguous local government authority areas in the lower North Island of New Zealand, all for signs within 10 metres of the playground equipment)

Territorial Local Authority	Play-grounds (N)	Smokefree signs		Dog control signs	
		Any signs	Mean no. of signs	Any signs	Mean no. of signs
Carterton District	2	50%	2.0	0%	0.0
Central Hawkes Bay District	2	50%	0.5	0%	0.0
Hastings District	3	67%	0.7	0%	0.0
Horowhenua District	2	50%	1.5	0%	0.0
Kapiti Coast District	2	0%	0.0	50%	0.5
Lower Hutt City	4	0%	0.0	75%	0.8
Manawatu District	2	0%	0.0	0%	0.0
Masterton District	2	100%	3.5	50%	1.0
Napier City	2	100%	1.5	50%	0.5
New Plymouth City	3	33%	0.3	0%	0.0
Palmerston North City	5	80%	1.0	60%	0.8
Porirua City	4	50%	0.5	0%	0.0
Rangitikei District	2	0%	0.0	0%	0.0
South Taranaki District	2	0%	0.0	0%	0.0
South Wairarapa District	2	100%	3.0	100%	2.5
Stratford District	2	50%	1.0	50%	0.5
Tararua District	2	0%	0.0	50%	0.5
Upper Hutt City	3	33%	0.3	0%	0.0
Wairoa District	2	50%	0.5	0%	0.0
Wellington City	13	38%	0.5	46%	0.5
Whanganui District	2	100%	2.5	50%	1.5
**Total/overall mean**	**63**	**44%**	**0.8**	**32%**	**0.4**

Figure A1: A relatively small smokefree sign by a playground (10 cm x 10 cm in size) and a similarly sized, but more obvious, dog control sign next to it

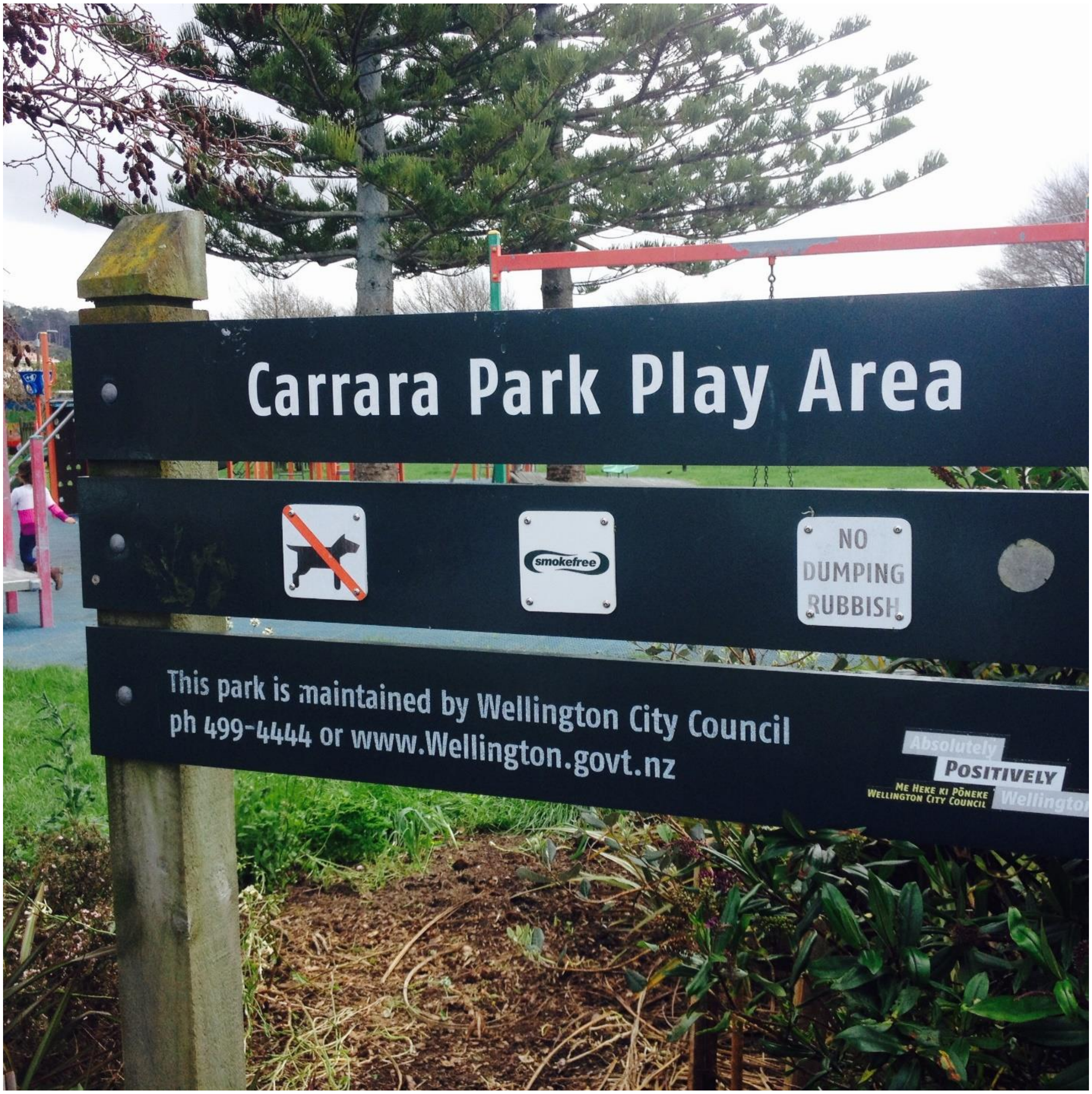

Figure A2: Contrasting dog control and smokefree signs with the dog control sign having: (i) a larger clearer image; (ii) fewer words; and (iii) reference to fines

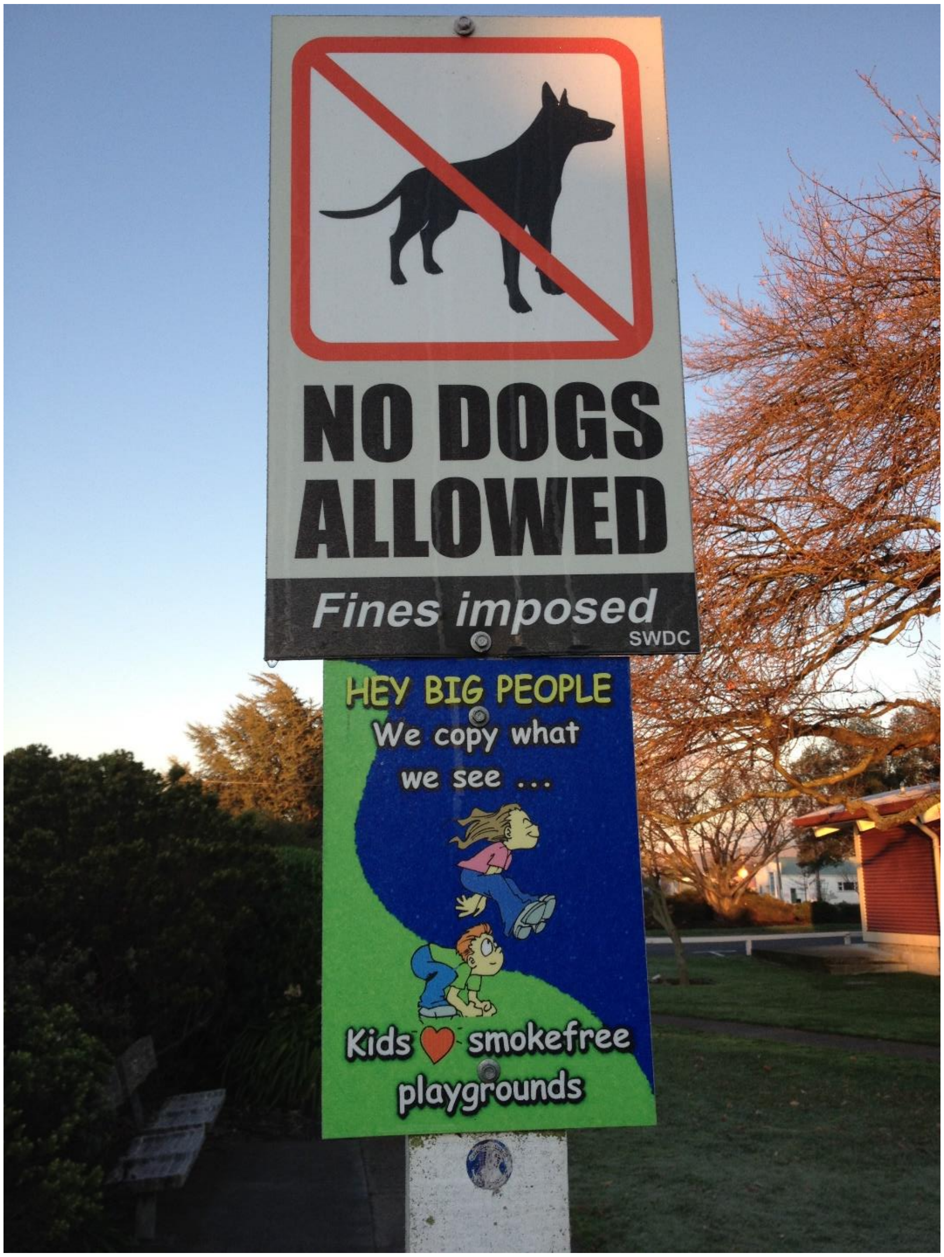

Figure A3: A smokefree sign message (Porirua City)

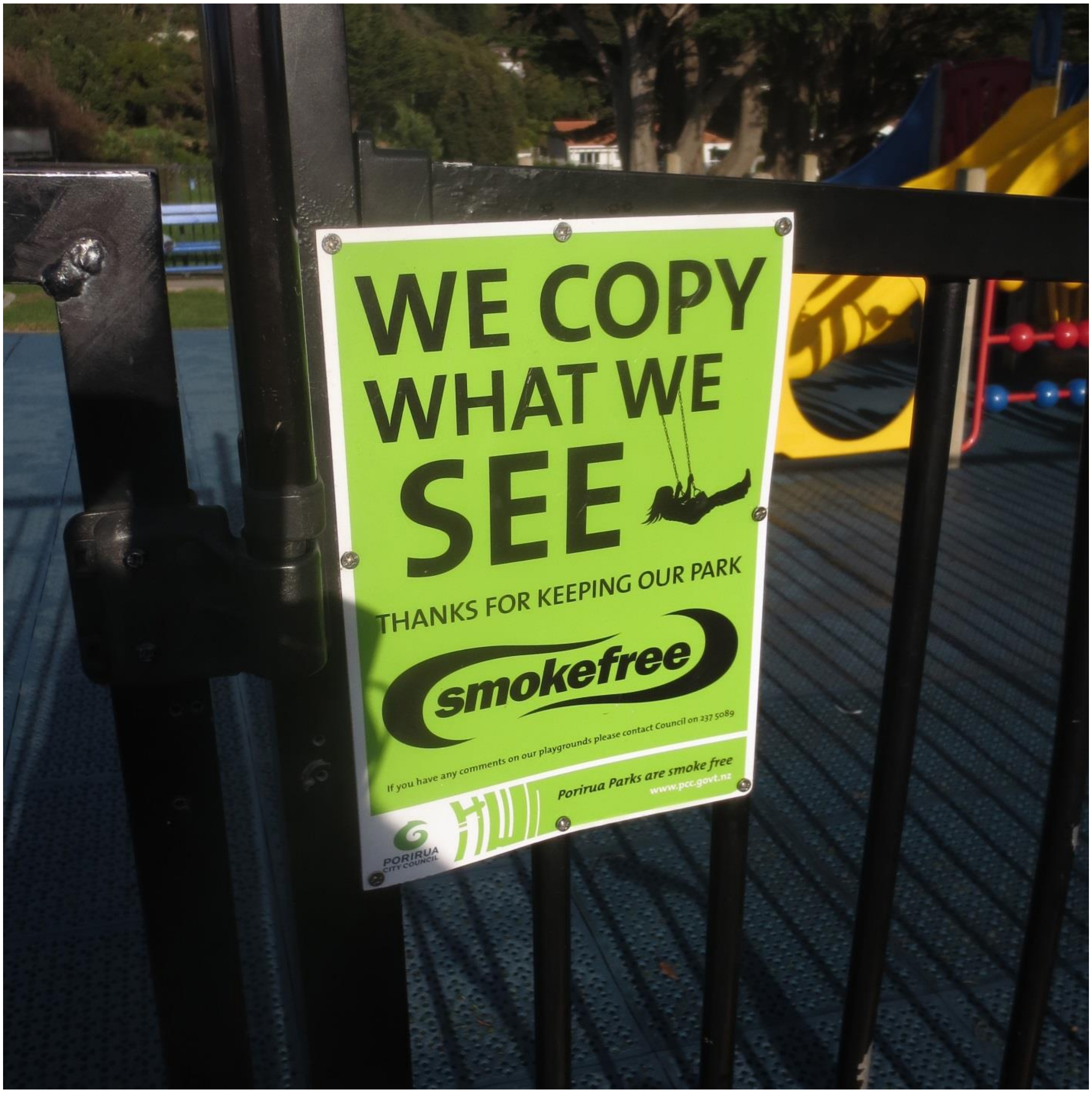

Figure A4: A relatively large smokefree sign by a playground that includes some Māori (indigenous) language and uses persuasive messaging

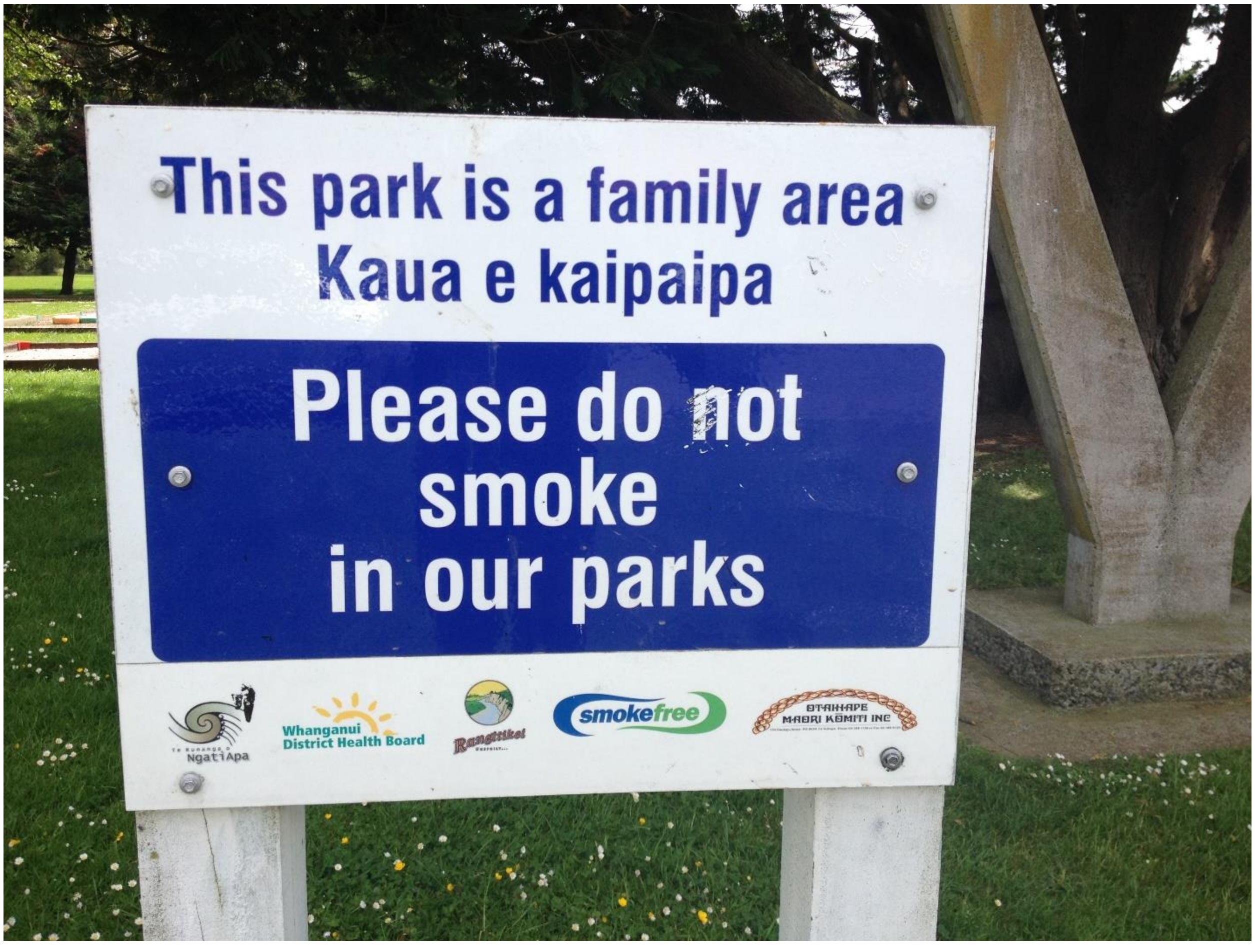

Supplement: Additional file 1: — Online appendix. (PDF 1085 kb) [file 12971_2017_143_MOESM1_ESM.pdf]
